# A representative metalloprotease induces PGE_2_ synthesis in fibroblast-like synoviocytes via the NF-κB/COX-2 pathway with amplification by IL-1β and the EP4 receptor

**DOI:** 10.1038/s41598-020-59095-z

**Published:** 2020-02-24

**Authors:** Mariana N. Viana, Elbio Leiguez, José M. Gutiérrez, Alexandra Rucavado, Regina P. Markus, Marina Marçola, Catarina Teixeira, Cristina M. Fernandes

**Affiliations:** 1 Pharmacology Laboratory, Butantan Institute, São Paulo, SP Brazil; 2 Clodomiro Picado Institute, School of Microbiology, University of Costa Rica, San José, Costa Rica; 3 Department of Physiology, Institute of Biosciences, São Paulo University, São Paulo, SP Brazil

**Keywords:** Inflammation, Rheumatic diseases

## Abstract

Inflammatory joint conditions are characterized by synovial inflammation, which involves activation of fibroblast-like synoviocytes (FLSs) and production of inflammatory mediators and matrix metalloproteases (MMPs) in joints. This study showed that the snake venom metalloprotease (SVMP) BaP1 activates FLSs to produce PGE_2_ by a mechanism dependent on COX-2, mPGES-1 and iPLA_2_s. BaP1 also induces IL-1β release, which up-regulates the production of PGE_2_ at a late stage of the stimulation. Expression of COX-2 and mPGES-1 are induced by BaP1 via activation of NF-κB pathway. While NF-κB p50 and p65 subunits are involved in up-regulation of COX-2 expression, only p65 is involved in BaP1-induced mPGES-1 expression. In addition, BaP1 up-regulates EP4 receptor expression. Engagement of this receptor by PGE_2_ triggers a positive feedback loop for its production by up-regulating expression of key components of the PGE_2_ biosynthetic cascade (COX-2, mPGES-1 and the EP4 receptor), thus contributing to amplification of BaP1-induced effects in FLSs. These data highlight the importance of FLS as a target for metalloproteases in joint inflammation and provide new insights into the roles of MMPs in inflammatory joint diseases. Moreover, our results may give insights into the importance of the catalytic domain, of MMPs for the inflammatory activity of these enzymes.

## Introduction

Inflammatory arthropathies have long been a leading cause of morbidity and disability, affecting many people worldwide^1^. These inflammatory joint conditions are characterized by different levels of chronic inflammation, invasive synovitis, destruction of cartilage and bone, and narrowing of the joint cavity^2^. Synovitis, which is associated with osteoarthritis and rheumatoid arthritis in other arthropathies, is characterized by chronic inflammatory changes in the joints involving different cell types and a wide range of inflammatory mediators. Fibroblast-like synoviocytes, which are also known as B-type synoviocytes, are key cells implicated in synovitis. They play a crucial role in driving persistent inflammation and articular damage by releasing various inflammatory mediators and take on an aggressive, invasive phenotype, leading to the breakdown of cartilage by matrix metalloproteases, which are found in high levels in synovial fluid during articular inflammatory processes^3–7^.

MMP family members are known for their ability to cleave components of the extracellular matrix, including collagens and adhesive glycoproteins^8,9^, as well as other non-matrix proteins. While playing important roles in physiological processes, these enzymes have been associated with the pathogenesis of various inflammatory diseases. Accordingly, the progressive destruction of cartilage and bone observed in inflammatory arthropathies has been shown to result from an imbalance between the levels of activated MMPs and tissue inhibitors of MMPs^10^. In addition, the biological actions of some MMPs, such as MMP-1, -2, -3 and -9, have been shown to result from their ability to modify proteolytically the activities of cytokine and chemokine precursors, growth factors and protease inhibitors in experimental models of inflammatory diseases^11^ Evidence from experimental models indicates that MMPs also actively participate in the development of inflammation and induction of hypernociception in articular joints^12^.

In addition to synthesizing MMPs, FLSs produce and secrete a wide range of proinflammatory mediators, including prostaglandins (PGs), and are the main cell source of prostaglandin E_2_ (PGE_2_) during inflammatory reactions in the joints^4,13^. PGE_2_, which is converted from arachidonic acid (AA) by the cyclooxygenases (COXs) enzymes and terminal PGE-synthases, plays a pivotal role in the pathogenesis of inflammatory arthropathies by mediating vasodilation, vascular permeability and pain^14,15^. This prostanoid has also been shown to modulate bone resorption by stimulating osteoclast formation from precursor stem cells, suggesting its involvement in tissue destruction, a characteristic of arthritic diseases^16^. It exerts its various effects by engaging four transmembrane receptors, subtypes EP1, EP2, EP3 and EP4. The PGE_2_-activated EP4 receptor signaling pathway is involved in the generation of articular pain and inflammation^17^. Furthermore, PGE_2_ has been detected in high concentrations in the synovial fluid, synovial membrane and serum of patients with rheumatoid arthritis and osteoarthritis, together with MMPs and other inflammatory mediators^18^.

Although joint inflammation is a complex reaction facilitated by several factors, such as proinflammatory lipid mediators, only a few studies have investigated which factors contribute more significantly to the production of prostaglandins such as PGE_2_ and consequent synovitis and hyperalgesia. MMP-1 and -3 have been reported to induce the release of PGE_2_ from macrophages *in vitro*^19^, but the regulatory role of MMPS in the production of PGE_2_ by FLSs has not been explored to date. As these cells are the main source of PGE_2_ in inflammatory arthropathies, this issue warrants further study.

SVMPs share structural and functional features with mammalian MMPs and have been grouped within the family of ‘metzincins’^20^. Like MMPs, SVMPs such as BaP1 from *Bothrops asper* snake venom can (i) degrade extracellular matrix components, (ii) induce inflammatory events and (iii) activate inflammatory cells^12,21–24^, and are therefore useful tools for studies on the biological effects of MMPs.

In light of this, we investigated the ability of BaP1 to induce the release of PGE_2_ from isolated FLSs and the molecular mechanisms involved in this effect. We also investigated the participation of COX-1 and -2, microsomal prostaglandin E synthase-1 (mPGES-1), nuclear factor kappa B (NF-κB), intracellular phospholipases A_2_ (PLA_2_s) and the EP4 receptor in BaP1-induced release of PGE_2_. Our results provide the first evidence that a metalloprotease can directly activate FLSs to release this prostanoid. This effect involves the COX-2/mPGES-1 pathway, which is mediated by NF-κB and modulated by a positive feedback loop triggered by engagement of the EP4 receptor.

## Material and Methods

### Animals

Male Wistar rats (190–220 g) were housed in temperature-controlled rooms and received food and water *ad libitum* until they were killed for the experiments. Animal procedures had been approved by the Institutional Animal Care and Use Committee of Butantan Institute (CEUAIB protocol number: 576/09) where animals received humane care according to the criteria outlined in the “Guide for the Care and Use of Laboratory Animals”^25^. Animal experiments complied with the ARRIVE guidelines and were carried out in accordance with the U.K. Animals (Scientific Procedures) Act, 1986 and associated guidelines, EU Directive 2010/63/EU for animal experiments, or the National Institutes of Health guide for the care and use of Laboratory animals (NIH Publications No. 8023, revised 1978).

### Venom metalloprotease

The metalloprotease BaP1 was isolated from a venom pool obtained from more than 40 adult specimens of *Bothrops asper* collected in the Pacific region of Costa Rica and kept at the serpentarium of the Clodomiro Picado Institute in Costa Rica. BaP1 was purified by ion-exchange chromatography on CM-Sephadex C-25, gel filtration on Sephacryl S-200 and Affi-Gel Blue affinity chromatography, and the homogeneity of the enzyme was confirmed by sodium dodecyl sulphate-polyacrylamide gel electrophoresis (SDS-PAGE) under reducing conditions^21,26^. The enzyme was lyophilized, stored at −20 °C and dissolved in Roswell Park Memorial Institute 1640 (RPMI) medium just before use. The default of endotoxin lipopolysaccharides (LPS) contamination in the BaP1 preparation was demonstrated by the quantitative Limulus amebocyte lysate (LAL) test^27^, which revealed undetectable levels of endotoxin (<0.125 EU/mL).

### Cell Culture

Synovial tissues were obtained from rat knee joints, and FLSs were isolated from these tissues as described previously^28^. Briefly, synovial tissues were digested for 4 h with 4 mg/mL collagenase (type IV: Sigma Aldrich, USA) in RPMI 1640 medium (Gibco, USA) at 37 °C in 5% CO_2_. The dissociated cells were plated in 12-well plates containing RPMI supplemented with 10% fetal bovine serum (FBS) (Gibco, USA), gentamycin (40 μg/mL) and L-glutamine (2 mM). When the cells reached full confluence they were harvested with 0.05% trypsin (Gibco, USA) and transferred into 25 to 175 cm^2^ flasks. Monolayers of FLSs were obtained by seeding different number of cells into 96- or 6-well plates depending on the experiments being carried out. Third- to ninth-passage cells were used in all experiments. The decision to use cells from these passages was based on the results of immunostaining assays showing that FLSs are the predominant cells at these passages, as indicated by the positive immunostaining for CD90, a marker of FLSs in these cells (Supplementary Information).

### Immunodetection of CD90

Immunostaining of CD90 was performed to detect FLSs. In brief, the cells were attached to coverslips and incubated with RPMI for 48 h, fixed with 2% paraformaldehyde, permeabilized with 0.2% Triton-X 100 in 0.1 M phosphate buffered saline (PBS) and blocked with 0.5% normal donkey serum in 0.1 M PBS for 1 h. After PBS washes, the cells were incubated for 1 h with anti-mouse CD90 (1:1000) diluted in 0.1 M PBS with 0.2% Triton-X 100. After three washes with PBS (5 min each), the cells were incubated for 1 h in the dark with secondary FITC-conjugated goat anti-mouse antibody (1:4000). After PBS washes, the slides were incubated with DAP1, a fluorescent dye (1:200), in 0.1 M PBS. After three washes, the slides were mounted with fluoromount G and examined under a confocal laser scanning microscope (Zeiss LSM 510 Meta).

### Stimulation and treatment of FLSs

FLSs were plated on glass coverslips in 6-well plates at a density of 7 × 10^5^ cells/coverslip and allowed to attach to the coverslips for 24 h in RPMI supplemented with 10% FBS, gentamycin (40 μg/mL) and L-glutamine (2 mM) at 37 °C, 5% CO_2._ Non-adherent cells were removed by washing with PBS. Cell monolayers were incubated with serum-free RPMI for 2 h to allow cells to achieve quiescence and were then challenged with BaP1 or medium (control). All pharmacological inhibitors were added between 60 and 120 min before stimulation of FLSs with BaP1 or medium (control). Stock solutions of COX-1, COX-2, NF-κB and EP4 inhibitors were prepared in dimethyl sulfoxide (DMSO) and stored at −20 °C and interleukin-1β (IL-1β) antagonist was prepared in saline solution and stored at 4 °C. Aliquots were diluted in RPMI to the required concentration immediately before use. The final DMSO concentration was always lower than 1% and had no effect on PGE_2_ release or COX-2 or mPGES-1 protein expression induced by BaP1. Listed below are the compounds and concentrations used as well as the treatment time of cells prior to the addition of BaP1: (I) Valeryl salicylate (Cayman Chemicals, USA), 1 h before and concomitantly to BaP1; (II) NS398 (Cayman Chemicals, USA), 1 h before and concomitantly to BaP1; (III) AH23848 (Cayman Chemicals, USA), 1 h before and concomitantly to BaP1; (IV) N-tosyl-L-phenylalanine chloromethyl ketone (TPCK, Sigma Aldrich Co., USA), 1 h before and concomitantly to BaP1; (V) specific NFκB inhibitor peptide (SN50, Calbiochem, GE), 1 before and concomitantly to BaP1 and (VI) Anakinra (Kineret) (Amgen Inc., Stockholm, Sweden), 2 h before and concomitantly to BaP1.

### Cytotoxicity assay

Cytotoxicity to FLSs of BaP1 and the inhibitors used in this study was assessed using the (3-(4,5-dimethylthiazol-2-yl)-2,5-diphenyltetrazolium bromide) tetrazolium reduction MTT assay. In brief, 1 × 10^4^ cells/well in RPMI-1640 medium supplemented with 2 mM L-glutamine were plated in 96-well plates and incubated with different concentrations of BaP1 (6.25, 12.5 and 25 µg/mL in culture medium) or with the same volume of culture medium alone (control) for 6, 12 and 24 h at 37 °C in a humidified atmosphere with 5% CO_2_. MTT (5 mg/mL) was dissolved in PBS and sterilized by filtration so that any insoluble residues were removed. Stock MTT solution (10% in culture medium) was added to all the wells in each experiment, and plates were incubated at 37 °C for 3 h. DMSO (100 µL) was then also added to all the wells, and the plates were stirred at room temperature for 30 min to dissolve any residual crystals. Absorbances were then recorded in a microtiter plate reader at 540 nm. Results were expressed as the percentage of viable cells, considering the control cells incubated with medium alone as 100% viable. No significant changes in cell viability were registered with any inhibitors or vehicle at the concentrations used (Supplementary Information).

### Determination of PGE_2_

The concentrations of PGE_2_ in cell culture supernatants were determined with the PGE_2_ enzyme-linked immunosorbent assay (ELISA) kit (monoclonal) (Cayman Chemical, USA). Briefly, 7 × 10^5^ cells in 6-well plates were treated with or without BaP1 (12.5 µg/mL). After 30 min, 1, 3, 6, 12 and 24 h the supernatants were collected and PGE_2_ was quantified according to the manufacturer’s protocol. Concentrations were calculated against a standard curve.

### Western blotting analysis

Protein expression of COX-1, COX-2, mPGES-1 and the PGE_2_ receptor EP4 was analyzed by western blotting. A total of 7 × 10^5^ cells grown in 6-well plates and stimulated or not with BaP1 (12.5 µg/mL) were lysed with 80 µL of sample buffer (0.5 M Tris-HCl, pH 6.8, 20% SDS, 1% glycerol, 1 M β-mercaptoethanol, 0.1% bromophenol blue) and boiled for 10 min. Samples were resolved by sodium dodecyl sulfate polyacrylamide gel electrophoresis (SDS-PAGE) on a 10% bis-acrylamide running gel overlaid with a 5% stacking gel. Proteins were then transferred to nitrocellulose membranes (GE Healthcare, Buckinghamshire, UK) with a Mini Trans-Blot Cell (Bio-Rad Laboratories, Richmond, CA, USA). The membranes were blocked for 1 h with 5% nonfat dry milk in Tris-buffered saline and Tween 20 (TTBS) (20 mM Tris, 100 mM NaCl and 0.5% Tween 20) and incubated with primary antibodies against COX-1 or COX-2 (for 1 h) and mPGES-1 or the EP4 receptor (for 18 h) diluted 1:1000, 1:500, 1:250 and 1:250, respectively (Cayman Chemical Company, Ann Arbor, MI, USA). An antibody against β-actin (1:2000) was used as a loading control (Sigma St Louis, MO, USA) and incubated for 1 h. The membranes were then washed and incubated with the appropriate secondary antibody conjugated to horseradish peroxidase. The enhanced chemiluminescence (ECL) reagent (GE Healthcare, Buckinghamshire, UK) was used to detect the proteins following the manufacturer’s instructions. Band densities were determined with a GS 700 Densitometer^®^ (Bio Rad Laboratories, Richmond, CA, USA) and Molecular Analyst^®^ (Bio-Rad Laboratories Richmond, CA, USA).

### Quantitative real-time RT-PCR and data analysis

Gene expression in FLSs was measured by quantitative real-time RT-PCR (qRT-PCR) using Platinum^TM^ *SYBR Green* qPCR SuperMix (Invitrogen^TM^) as the reporter fluorophore to measure mRNA levels. GAPDH mRNA levels were used to normalize the amount of total RNA in each reaction. The results were normalized using Ct-values (the relative threshold cycle method) (Ct values of mRNA levels in stimulated cells minus Ct values of a given gene in resting cells). Primer sequences (5′ to 3′ orientation) were as follows: for COX-2 (10 pmol/mL) forward (CAT GAT CTA CCC TCC CCA CG, COX-2) and reverse (CAG ACC AAA GAC TTC CTG CCC); for mPGES-1 (10 pmol/mL) forward (GCG AAC TGG GCC AGA ACA) and reverse (GGC CTA CCT GGG CAA AAT); and for GAPDH forward (AAG AAG GTG GTG AAG CAG GC) and reverse (TCC ACC ACC CTG TTG CTG TA).

### Preparation of nuclear extracts and electrophoretic mobility shift and supershift assays (EMSA)

FLSs were treated with medium or BaP1 for 30 or 60 min, and nuclear extracts were prepared as described previously with minor modifications^29^. Briefly, the cells were resuspended in PBS and centrifuged (2,500 g, 10 min, 4 °C). Cell pellets were homogenized with 200 μL lysis buffer (10 mm HEPES pH 7.5, 10 mm KCl, 0.1 mm EDTA pH 8.0, 10% glycerol, 1 mm DTT, 0.1 mm PMSF) plus 7 μL NP-40 (10%). The samples were homogenized for 10 s, maintained on ice for 15 min and then centrifuged (12,000 g, 1 min, 4 °C). The pellets were washed gently with 100 μL lysis buffer and centrifuged (12,000 g, 1 min, 4 °C). Nuclear extract buffer (10 mm HEPES pH 7.5, 500 mm KCl, 1 mm EDTA pH 8.0, 10% glycerol, 1 mm DTT, 0.1 mm PMSF) was added to the nuclear pellets, and samples were incubated on ice under shaking (15 min) and centrifuged (20,000 g, 5 min, 4 °C). Supernatants containing the nuclear proteins were collected in clean microtubes and stored at −20 °C. Total nuclear protein concentration was quantified at 495 nm using a ND-1000 spectrophotometer (Nanodrop Technologies, Wilmington, DE, USA). The double-strand consensus NF-κB oligonucleotide probe (5′-AGTTGAGGGGACTTTCCCAGGC-3′) (Sinapse, São Paulo, SP, Brazil) was end-labeled with [γ32P]-ATP (specific activity 3000 Ci/mmol) in the presence of T4 polynucleotide kinase (10 min, 37 °C). A total of 6 μg of nuclear protein was incubated with binding buffer (10 mm Tris-HCl pH 7.5, 1 mm MgCl2, 50 mm NaCl, 0.5 mm DTT, 0.5 mm EDTA, 4% glycerol) plus 1.0 μL of poly (dI-dC) (20 min, room temperature) to a final volume of 20 µL. The samples were incubated with ∼40,000 counts/min of radiolabeled probe (30 min, room temperature). The protein-DNA complexes were analyzed on nondenaturing 6% polyacrylamide gels at 150 V for 1 h 30 min in TBE buffer (0.25X Tris-Borate-EDTA). The gel was vacuum-dried and exposed to XAR-5 Kodak film (Rochester, NY, USA) for 48–72 h at −70 °C. Autoradiograms were scanned and analyzed by densitometry with ImageJ (http://rsb.info.nih.gov/ij). A supershift assay was performed to identify the NF-κB subunits. To this end, samples were incubated with 2 μL of rabbit polyclonal affinity-purified antibodies(sc-109×, sc-114×, sc-298×, sc-70x and sc-226×) against each NF-κB subunit (RelA, p50, p52, c-Rel and RelB, respectively) (Santa Cruz Biotechnology) for 45 min at room temperature before adding the γ32P-labeled-NF-κB probe.

### Statistical analysis

Data are expressed as standard error of the mean (SEM) of at least three independent experiments. Multiple comparisons between groups were performed by one-way one-way analysis of variance (ANOVA) followed by Tukey’s test. Probabilities of less than 5% (p < 0.05) were considered significant.

## Results

### Metalloprotease BaP1 induces PGE_2_ release by cultured FLSs

**Figure 1:**
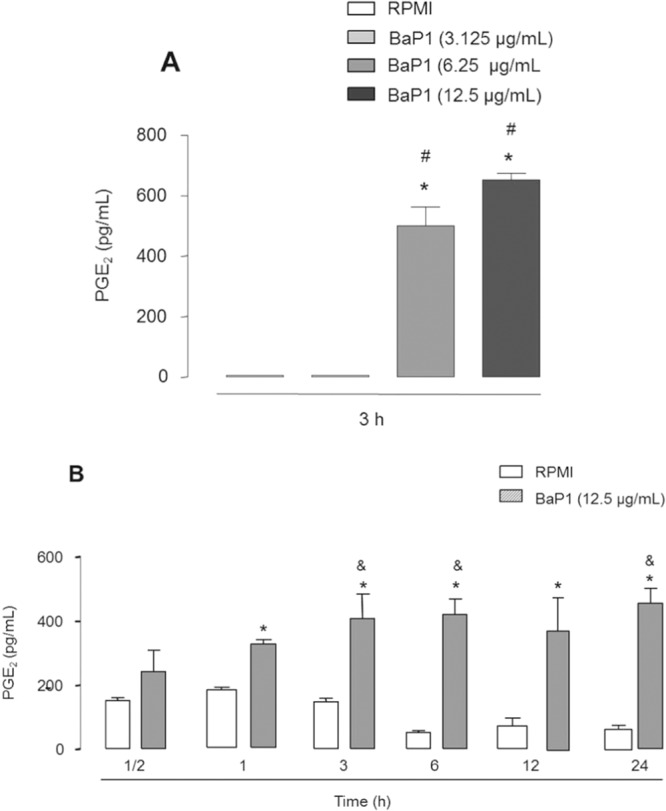
BaP1 induces production of PGE_2_ in FLSs. (**A**) Effect of different concentrations of BaP1 on PGE_2_ release in FLSs incubated with various concentrations of BaP1 or RPMI (control) for 3 h. (**B**) Time-course of BaP1-induced PGE_2_ release. FLS monolayers were incubated with BaP1 (12.5 µg/mL) or RPMI (control) for 30 min, 1, 3, 6, 12 and 24 h. Supernatants were collected, and PGE_2_ was quantified by ELISA. Results are expressed as mean ± SEM of 4 samples. *p < 0.05 relative to control; ^**#**^p < 0.05 relative to BaP1 (3.125 µg/mL); ^**&**^p < 0.05 relative to BaP1 (1 h) (one-way ANOVA, Bonferroni post test in A and two-way ANOVA, Bonferroni post test in B).

PGE_2_ is recognized as a key mediator in the pathogenesis of inflammatory arthropathies^30,31^.The release of this prostanoid from macrophages has been reported to be induced by MMPs, which are present in high levels in inflamed articular joints^19^. However, whether this class of enzymes stimulates FLSs to produce PGE_2_ has yet to be established. Therefore, to investigate the possible action of metalloproteases in FLSs, we assessed the ability of BaP1 to induce the release of PGE_2_ in cultured FLSs, by testing three concentrations of BaP1 (3.125, 6.25 and 12.5 μg/mL) added to the culture for 3 hours. At these concentrations, BaP1 did not affect cell viability after 6, 12 or 24 h of incubation as assessed by the MTT assay (Fig. S1). As shown in Fig. 1A, at concentrations of 6.25 and 12.5 μg/mL, but not 3.125 μg/mL, BaP1 induced significant release of PGE_2_ from FLSs compared with control cells incubated with culture medium alone. After establishing that BaP1 induced a maximal effect at 12.5 μg/mL, we evaluated the time course of BaP1-induced PGE_2_ release. As shown in Fig. 1B, increased levels of PGE_2_ were detected from 1 to 24 h after stimulation with BaP1, indicating that BaP1 can directly stimulate FLSs to produce PGE_2_ and supporting previous findings by our group that an intra-articular injection of BaP1 into the rat knee joint results in increased levels of PGE_2_ in the synovial fluid^12^. These results strongly suggest that FLSs are important targets for metalloprotease-induced PGE_2_ production during joint inflammation.

### COX-1 and COX-2 contribute to BaP1-induced PGE_2_ release

**Figure 2:**
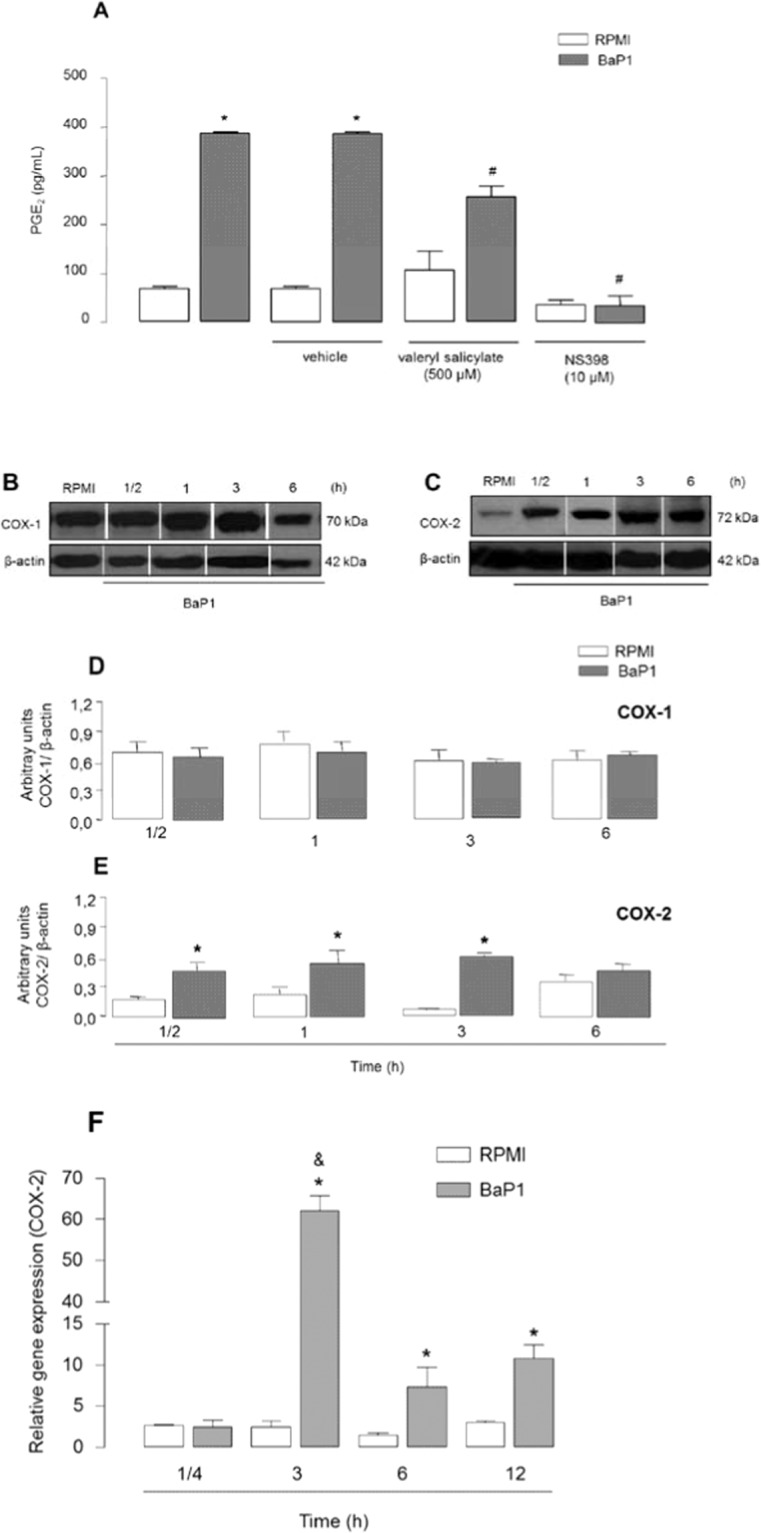
BaP1 activates COX-1 and -2 pathways to release PGE_2_ and up-regulates COX-2 expression. (**A**) Isolated FLSs were pretreated with the selective inhibitors of COX-1 and -2, valeryl salicylate and NS398, respectively, for 1 h and incubated with BaP1 (12.5 µg/mL) or RPMI (control) for 3 h. Supernatants were collected, and PGE_2_ was quantified by ELISA. (**B–E**) FLSs were incubated with BaP1 (12.5 µg/mL) or RPMI (control) for 30 min, 1, 3 and 6 h. (**B**) Representative Western blotting of COX-1 and β-actin (loading control) showing immunoreactive bands. (**C**) Representative Western blotting of COX-2 and β-actin (loading control) showing immunoreactive bands. Densitometric analysis of immunoreactive (**D**) COX-1 and (**E**) COX-2 bands. (**F**) COX-2 relative gene expression determined by quantitative PCR in cells incubated with BaP1 (12.5 µg/mL) or RPMI (control) for 15 min, 3 and 6 h. Data are mean ± SEM of 3 samples. *p < 0.05 relative to control; ^**#**^p < 0.05 relative to BaP1 (with vehicle); ^**&**^p < 0.05 relative to BaP1 (6 and 12 h) (one-way ANOVA, Bonferroni post test in A and two-way ANOVA, Bonferroni post test in D, E and F). Blots were cropped from the full-length blots, which are demonstrated as Supplementary Figures 3(A,B) and 3(A–D) and cropped blots are indicated by arrows.

To investigate the mechanisms underlying BaP1-induced PGE_2_ synthesis, we examined the participation of key enzymes, COX-1 and -2, in the PGE_2_ biosynthetic cascade. FLSs were treated for 1 h with valeryl salicylate or NS398, selective inhibitors of COX-1 and -2, respectively, or their vehicles. PGE_2_ release was evaluated after 3 h of incubation with BaP1. As shown in Fig. 2A, FLSs pretreated with vehicle only and stimulated with BaP1 secreted greater amounts of PGE_2_ than basal FLSs (negative control). Pretreatment of FLSs with valeryl salicylate significantly decreased BaP1-induced PGE_2_ production in comparison with FLSs treated only with this inhibitor vehicle, without valeryl salicylate and without BaP1. Preincubation of cells with NS398 also abolished PGE_2_ release induced by BaP1 (Fig. 2A). These results suggest that both COX-1 and -2 play an important role in BaP1-induced PGE_2_ production in FLSs. Having shown that the inducible COX-2 isoform participates in the signaling pathway triggered by BaP1 that leads to PGE_2_ production, we next investigated whether BaP1 is able to induce protein expression of COX-2 in FLSs. Our results show that FLSs spontaneously expressed COX-1 and -2. COX-1 protein expression did not differ significantly between control cells and cells treated with BaP1 (Fig. 2B,C). However, protein expression of COX-2 was higher in cells incubated with the metalloprotease (30 min-3 h) (Fig. 2B,D). To examine whether BaP1-induced COX-2 protein expression is the result of continuous transcription, we evaluated gene expression of this isoform in FLSs stimulated with BaP1. COX-2 gene expression was enhanced in cells incubated with BaP1 after 1 and 3 h of incubation compared with control cells (Fig. 2E). These data indicate that BaP1 triggers transcription mechanisms in FLSs that result in an increase in COX-2 protein content.

### BaP1-induced mPGES-1 protein and gene expression

**Figure 3:**
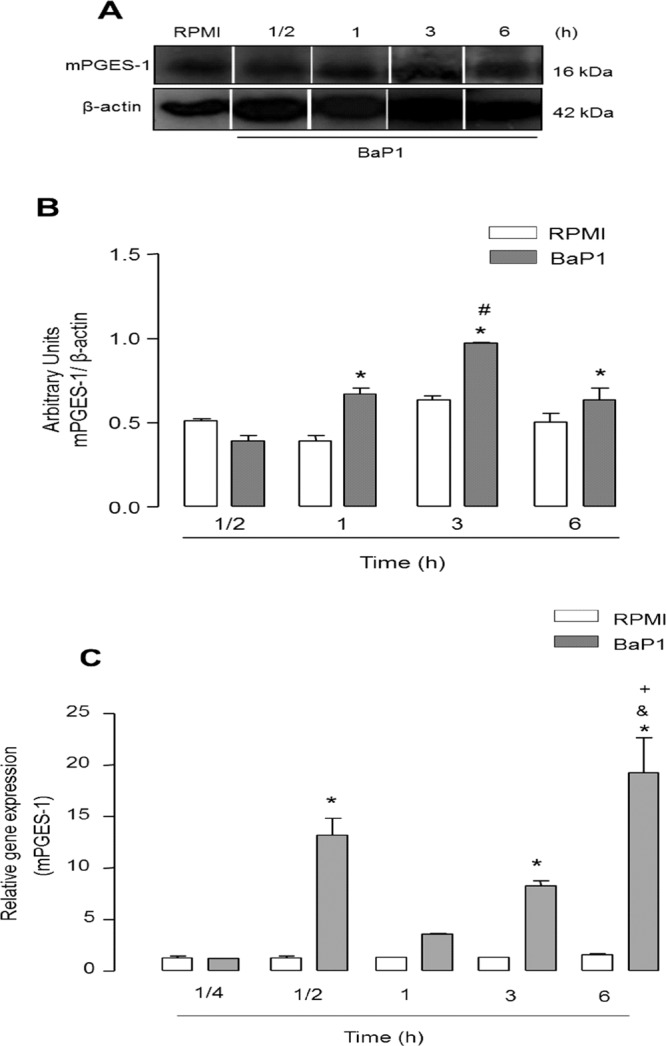
BaP1 up-regulates mPGES-1 protein and gene expression in FLSs. FLSs were incubated with BaP1 (12.5 µg/mL) or RPMI (control) for 15, 30 min, 1, 3 and 6 h. (**A**) Representative Western blotting of mPGES-1 and β-actin (loading control) showing immunoreactive bands. (**B**) Densitometric analysis of immunoreactive mPGES-1 bands. (**C**) Relative gene expression in cells incubated with BaP1 and control cells determined by quantitative PCR. Densities (in arbitrary units) were normalized to β-actin densities. Results are expressed as mean ± SEM of 4–5 samples. *p < 0.05 relative to control; ^**#**^p < 0.05 relative to BaP1 (1 h); ^**&**^p < 0.05 relative to BaP1 (1/2 h); ^**+**^p < 0.05 relative to BaP1 (3 h) (two-way ANOVA, Bonferroni post test). Blots were cropped from the full-length blots, which are demonstrated as Supplementary Figure 5(A–H) and cropped blots are indicated by arrows.

As mPGES-1 is an inducible enzyme responsible for the final step in the PGE_2_ biosynthetic cascade, we further explored the mechanisms involved in BaP1-induced PGE_2_ production in FLSs by assessing mPGES-1 gene and protein expression after stimulation with BaP1. As shown in Fig. 3A,B, the increase in mPGES-1 protein expression in BaP1-stimulated cells after 3 and 6 h incubation was greater than in the non-stimulated control cells. Analysis of mPGES-1 gene expression after stimulation with BaP1 revealed increased levels of mPGES-1 mRNA after 3 and 6 h of stimulation (Fig. 3C). These results highlight a mechanism by which BaP1 induces PGE_2_ biosynthesis and suggest that activation of mPGES-1 gene expression is the primary mechanism leading to an increase in this protein.

### Involvement of Intracellular PLA_2_s in BaP1-induced PGE_2_ release

**Figure 4:**
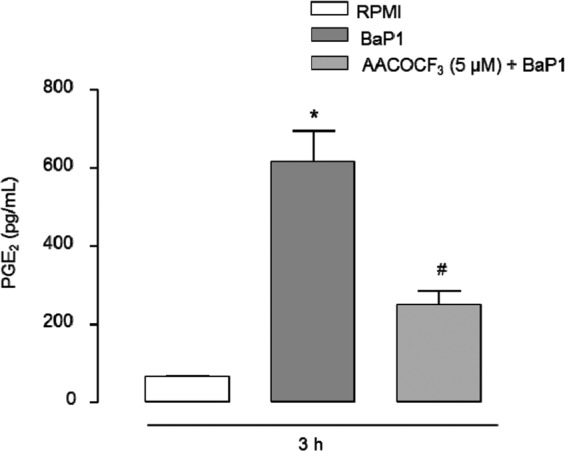
Intracellular PLA2s contribute to BaP1-induced PGE_2_ release. Isolated FLSs were pretreated with AACOCF3 (5 μM) for 1 hour and stimulated by BaP1 (12.5 mg/mL) or RPMI (control) for 3 h. Supernatants were collected and PGE_2_ was quantified by ELISA. Results are expressed as mean ± SEM of 4 samples. *p < 0.05 relative to control. ^#^p < 0.05 relative to BaP1 (one-way ANOVA, Bonferroni post test).

It is well known that PLA_2_s hydrolyze membrane phospholipids, resulting in the release of arachidonic acid, which is further converted by the COX enzymes and prostaglandin synthases into biologically active prostaglandins, such as PGE_2_. To investigate the role of intracellular PLA_2_s in PGE_2_ release, we checked the effect of arachidonyl trifluoromethyl ketone (AACOCF3), an intracellular inhibitor of PLA_2_s, on BaP1-induced PGE_2_ release. Figure 4 shows that AACOCF3 caused a reduction in PGE_2_ levels after 3 h of stimulation with BaP1, indicating that BaP1-induced PGE_2_ release is also mediated by intracellular PLA_2_s.

### BaP1-induced effects on FLSs are dependent on NF-κB

**Figure 5:**
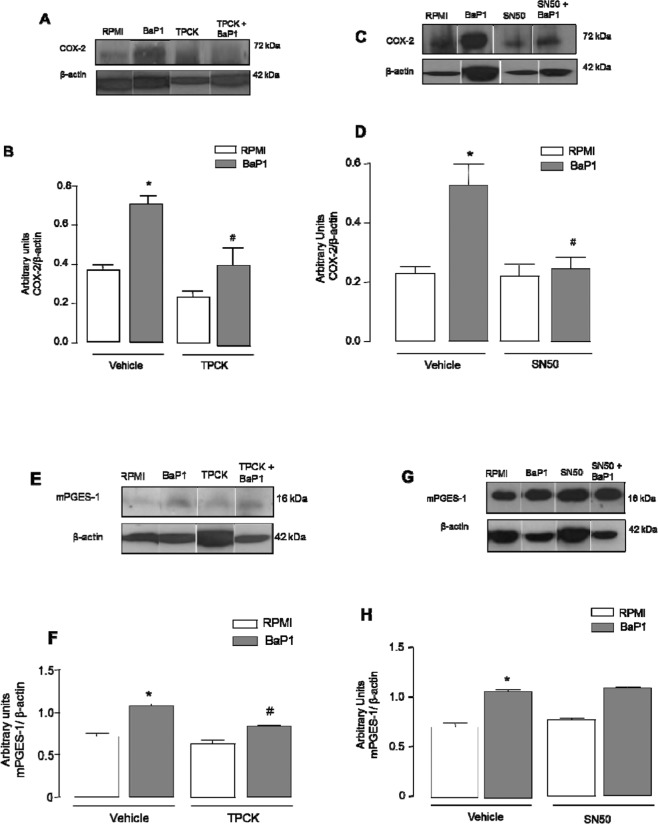
NF-kB transcription factor is relevant to BaP1-induced COX-2 and mPGES-1 protein expression by FLSs. Isolated FLSs were pretreated with TPCK (25 μM) or SN50 (50 μg/mL) for 1 hour and stimulated by BaP1 (12.5 mg/mL) or RPMI (control) for 3 h. (**A**,**C**) Representative Western blotting of COX-2 and β-actin (loading control) showing immunoreactive bands. (**B**,**D**) Densitometric analysis of immunoreactive COX-2 bands. (**E**,**G**) Representative Western blotting of mPGES-1 and β-actin (loading control) showing immunoreactive bands. (**F** and **H**) Densitometric analysis of immunoreactive mPGES-1 bands. (**E**) Results are expressed as mean ± SEM of 3–4 samples. *p < 0.05 relative to control. ^#^p < 0.05 relative to BaP1 (one-way ANOVA, Bonferroni post test). Blots were cropped from the full-length blots, which are demonstrated as Supplementary Figures 6(A–D) and 7(A–D) and cropped blots are indicated by arrows.

NF-κB is recognized as a major transcription factor that regulates many inflammatory genes, including COX-2 and mPGES-1^32–36^. We therefore investigated the involvement of this factor in BaP1-induced expression of both biosynthetic enzymes. Cells were pretreated for 1 h with TPCK compound, an inhibitor of NF-κB activation by inhibiting K-B phosphorylation, and BaP1 effects were assessed after 3 h of incubation. As shown in Fig. 5, pretreatment of cells with TPCK inhibited both BaP1-induced COX-2 protein expression (Fig. 5A,B) and BaP1-induced mPGES-1 protein expression (Fig. 5E,F). In another set of experiments, cells were pretreated for 1 h with SN50 compound, an inhibitor of the NF-κB p50 subunit, and BaP1 effects were assessed after 3 h of incubation. As shown in Fig. 5, pretreatment of cells with SN50 inhibited BaP1-induced COX-2 protein expression (Fig. 5C,D) and BaP1-induced mPGES-1 protein expression (Fig. 5G,H). Taken together, these results indicate that activation of the NF-κB pathway plays an important role in BaP1-induced expression of the inducible enzymes COX-2 and mPGES-1.

### BaP1 activates NF-κB

**Figure 6:**
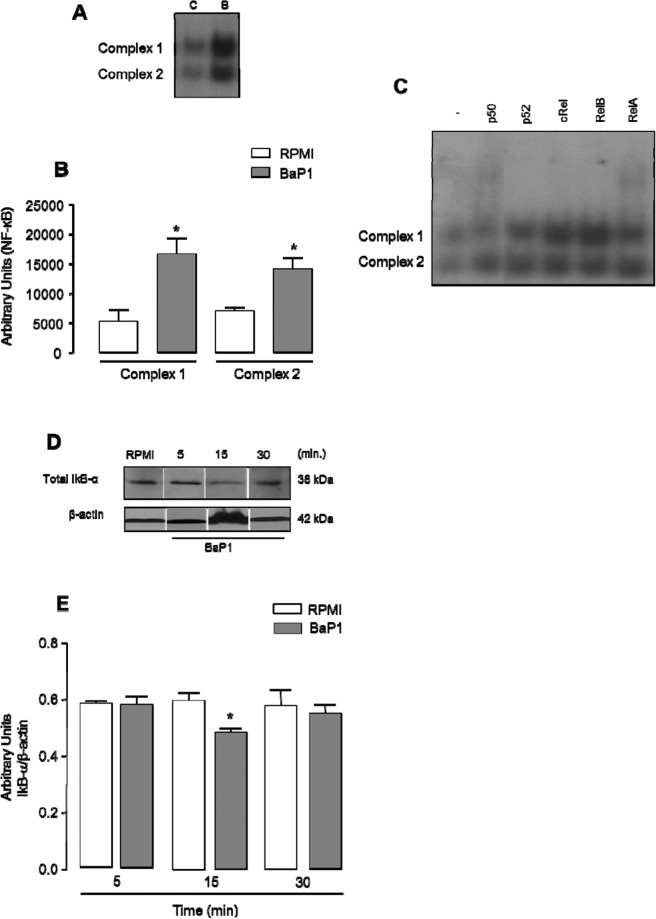
Effect of BaP1 on NF-kB activity in FLSs. (**A**) Cultured FLS were exposed to 12.5 μg/mL BaP1 for 30 and 60 min. Nuclear extracts were then prepared and analyzed by electrophoretic mobility shift assay (EMSA). (**B**) Densitometric analysis of immunoreactive NF-kB bands. (**C**) Binding specificity to detected bands was confirmed by supershift assays with p50, RelB, p52, cRel and RelA. (**D**) IkB-α degradation. FLSs were exposed to 12.5 μg/mL BaP1 for 5, 15 and 30 min. Representative Western blotting of IkB-α and β-actin (loading control) showing immunoreactive bands. (**E**) Densitometric analysis of immunoreactive IkB-α bands. Densities (in arbitrary units) were normalized to β-actin densities. Results are expressed as mean ± SEM of 3–4 samples. *p < 0.05 relative to control (one-way ANOVA, Bonferroni post test in B and two-way ANOVA, Bonferroni post test in E). Blots were cropped from the full-length blots, which are demonstrated as Supplementary Figures 8(A–D) and 11(A–C) and cropped blots are indicated by arrows.

To better understand the involvement of NF-κB in the effect of BaP1 on FLSs, the presence of NF-κB in the nucleus of FLSs incubated or not with BaP1 (12.5 µg/mL) was examined by EMSA. As seen in Fig. 6A, two bands representing two NF-κB complexes (C1 and C2) were observed in the control cells. Following 30 min incubation with BaP1, NF-κB protein expression was higher than in control cells (Fig. 6B). To identify the subunits present in the nucleus, a supershift assay was performed and the samples were incubated with antibodies that recognize the p50, Rel A, p52, c-Rel and Rel B subunits. As seen in Fig. 6C, only the p50 and Rel A subunits were displaced after incubation of samples with the antibodies against NF-κB subunits, demonstrating that these two subunits are present in the nucleus of FLSs. Moreover, the total protein content of the inhibitor of kappa B (IκB-α) had decreased after 15 min of incubation with BaP1, but remained unchanged in the control cells (Fig. 6D,E), confirming activation of NF-κB in FLSs stimulated with BaP1.

### EP4 receptor contributes to BaP1-induced effects

**Figure 7:**
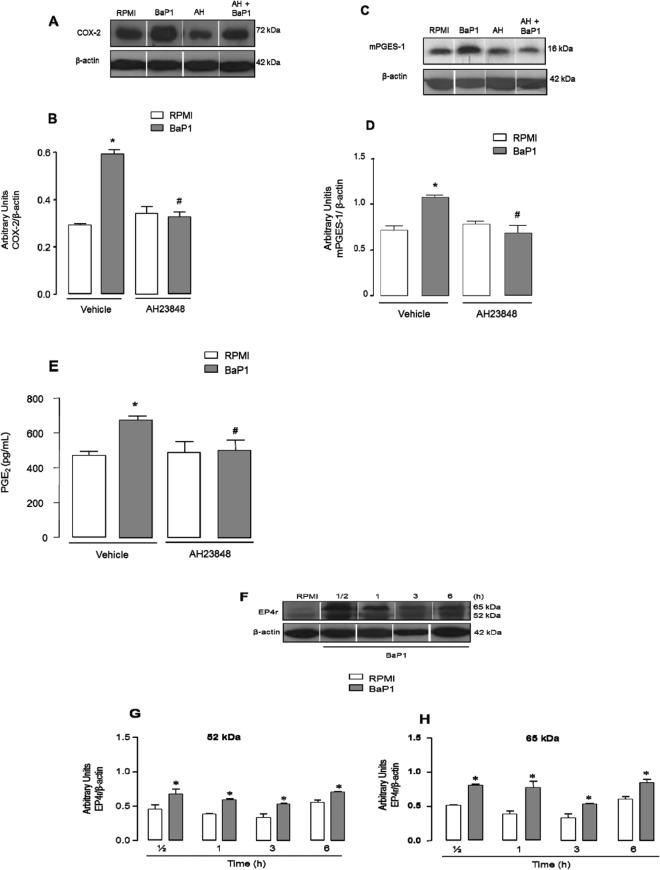
EP4 receptor antagonist blocks BaP1-induced effects in FLSs and BaP1up-regulates the expression of EP4 receptor. Isolated FLSs were pretreated with AH23848 (30 μM) and stimulated by BaP1 (12.5 mg/mL) or RPMI (control) for 3 hours. (**A**) Representative Western blotting of COX-2 and β-actin (loading control) showing immunoreactive bands. (**B**) Densitometric analysis of immunoreactive COX-2 bands. Densities (in arbitrary units) were normalized to β-actin densities. (**C**) Representative Western blotting of mPGES-1 and β-actin (loading control) showing immunoreactive bands. (**D**) Densitometric analysis of immunoreactive mPGES-1 bands. Densities (in arbitrary units) were normalized to β-actin densities. (**E**) PGE_2_ concentrations determined by ELISA. Results are expressed as mean ± SEM of 4 samples. (**F**) Representative Western Blotting of EP4 receptor and β-actin (loading control) showing immunoreactive bands. (**G**) Densitometric analysis of immunoreactive EP4 (54 kDa) bands. (**H**) Densitometric analysis of immunoreactive EP4 (62 kDa) bands. Densities (in arbitrary units) were normalized to β-actin densities. Data are mean ± SEM of 3 samples *p < 0.05 relative to control; ^#^p < 0.05 relative to BaP1 (one-way ANOVA, Bonferroni post test in B, D and E and two-way ANOVA, Bonferroni post test in G and H). Blots were cropped from the full-length blots, which are demonstrated as Supplementary Figures 9(A–D) and 10(A–D) and cropped blots are indicated by arrows.

In inflammatory joint diseases, the EP4 receptor is the major receptor subtype involved in PGE_2_ effects. As activation of this receptor by PGE_2_ can induce synthesis of other inflammatory proteins, including COX-2 and mPGES-1, we investigated the involvement of EP4 in BaP1-induced effects. To this end, FLSs were pretreated with AH23848, an EP4 receptor antagonist, and the BaP1-induced effects were evaluated after 3 hours of stimulation. Figure 7A,B shows that AH23848 significantly decreased BaP1-induced COX-2 protein expression in comparison with the control. Similarly, pretreatment of cells with the EP4 receptor antagonist significantly reduced mPGES-1 protein expression (Fig. 7C,D). As shown in Fig. 7E, PGE_2_ production was abolished when cells were pretreated with AH23848. These results indicate that engagement of the EP4 receptor by PGE_2_ regulates COX-2 and mPGES-1 expression and BaP1-induced production of PGE_2_. To better understand the involvement of the EP4 receptor in the effects induced by BaP1, we next analyzed EP4 receptor protein expression in FLSs stimulated with BaP1 and in control cells incubated with culture medium alone. As shown in Fig. 7F–H, there was a greater increase in EP4 receptor protein expression in both non-glycosylated (52 kD) and glycosylated (65 kDa) forms of the EP4 receptor in the cells stimulated with BaP1 at all incubation time points from 30 min to 6 h than in the control cells. These data reveal a route through which the pathway leading to PGE_2_ production in metalloprotease-stimulated FLSs is induced.

### IL-1β contributes to PGE_2_ production

**Figure 8:**
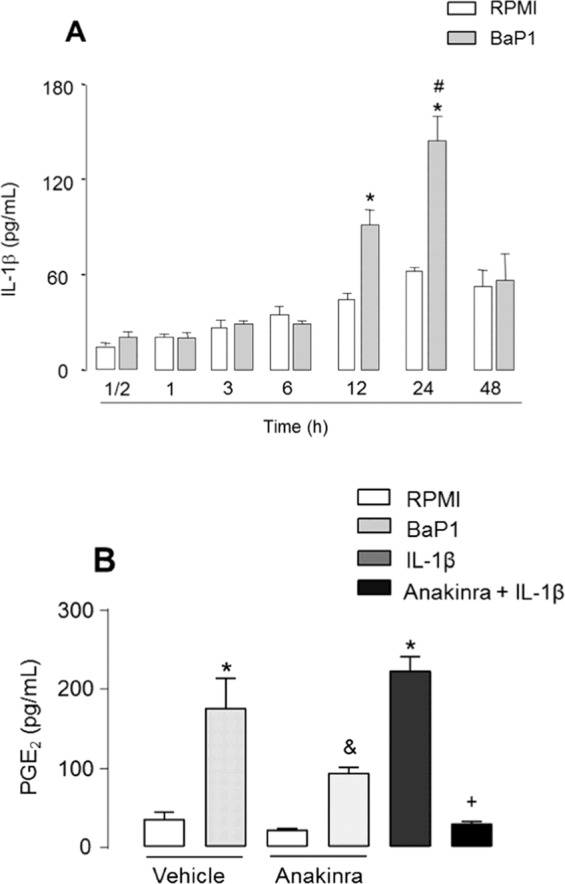
BaP1 induces production of IL-1β, which contributes to PGE_2_ production. (**A**) Time-course of BaP1-induced IL-1β release. FLS monolayers were incubated with BaP1 (12.5 µg/mL) or RPMI (control) for 30 min, 1, 3, 6, 12 and 24 h. (**B**) Isolated FLSs were pretreated with Anakinra (50 μM) or vehicle and stimulated by BaP1 (12.5 mg/mL) or RPMI (control) or IL-1β for 12 hours. Supernatants were collected, and IL-1β and PGE_2_ were quantified by ELISA. Results are expressed as mean ± SEM of 4 samples. *p < 0.05 relative to control. ^#^p < 0.05 relative to BaP1 (12 h); ^&^p < 0.05 relative to BaP1; ^**+**^p < 0.05 relative to IL-1β (two-way ANOVA, Bonferroni post test in A and one-way ANOVA, Bonferroni post test in B).

Proinflammatory cytokines such as IL-1β are produced in activated FLSs and are recognized as potent inducers of PGE_2_ biosynthesis in these cells. To determine the ability of BaP1 to induce the release of IL-1β, FLSs were incubated with BaP1 for different times. Significant production of IL-1β was observed after 12 h and 24 h of incubation in comparison with control cells (Fig. 8A). To explore the role of IL-1β in BaP1-induced PGE_2_ release, we examined the effect of anakinra, an IL-1β receptor antagonist, on FLSs stimulated with BaP1. As seen in Fig. 8B, pretreatment of FLSs with anakinra significantly reduced BaP1-induced PGE_2_ release after 24 h of incubation, indicating that IL-1β plays a role in BaP1-induced PGE_2_ production in FLSs.

## Discussion and Conclusions

Matrix metalloproteases are known to be important players in various inflammatory conditions, and high levels of these enzymes have been found in the synovial fluid of patients with inflammatory arthropathies^37,38^. Therefore, owing to the structural similarities between SVMPs and MMPs, investigation of the effects of the metalloprotease BaP1 on FLSs sheds light for understanding the role played by MMPs in the pathogenesis of these arthropathies. As shown in this study, FLSs in culture are directly activated by BaP1, leading to release of PGE_2_. This effect had a rapid onset and was sustained for up to 24 h after stimulation. Although the ability of MMP-1 and -3 to release PGE_2_ from isolated macrophages has been described previously in the literature^19^, to our knowledge this is the first report that provides evidence of a metalloprotease inducing production of PGE_2_ in FLSs. As these synoviocytes are a major source of PGE_2_ in joints and PGE_2_ is a crucial mediator of inflammatory arthropathies, our findings may help in the design of new therapeutic agents to treat inflammatory joint diseases. PGE_2_ is derived from the metabolism of arachidonic acid by cyclooxygenases enzymes followed by isomerization of the product prostaglandin H2 (PGH_2_) by terminal synthases^14^. Accordingly, our data show that BaP1 induced a marked increase in protein expression of both COX-2 and mPGES-1, the increase in COX-2 expression occurring before that of mPGES-1. In addition, the release of PGE_2_ correlated with increased expression of COX-2 and mPGES-1, indicating that these enzymes are involved in the BaP1-induced PGE_2_ biosynthetic cascade. This was confirmed by a pharmacological approach, which showed that COX-2 inhibitor abrogated BaP1-induced release of PGE_2,_ and implies functional coupling of COX-2 and mPGES-1 expression in the synthesis of this prostanoid. Furthermore, our findings that BaP1 induced gene expression of COX-2 and mPGES-1 demonstrate that BaP1 can trigger transcriptional mechanisms in FLSs that result in increased protein expression. Our results are in line with reports of increased levels of PGE_2_ in inflammatory processes in articular joints as a result of activation of the COX-2/mPGES-1 pathway^39,40^ and agree with those of Khan *et al*.^19^ showing increased mPGES-1 gene expression in macrophages stimulated with MMP-1 or -3.

As arachidonic acid, the main substrate of COXs enzymes, is released by the action of PLA_2_s on membrane glycerophospholipids, the role of this lipolytic enzyme in the effect of BaP1 was investigated. Our data demonstrating that pharmacological inhibition of intracellular PLA_2_s by AACOCF3 markedly reduced BaP1-induced release of PGE_2_ indicate that intracellular PLA_2_s are important players in the generation of PGE_2_ following BaP1 stimulus. This finding reveals for the first time a link between metalloproteases and intracellular PLA_2_s whereby the venom metalloprotease stimulates FLSs to produce PGE_2_. However, the mechanism, or mechanisms, by which BaP1 recruits the intracellular enzymes were not investigated here and deserve further studies.

Several works have established that nuclear factor NF-κB coordinates expression of inflammatory mediators and enzymes involved in the inflammatory response, including COX-2^41,42^. Furthermore, activation of this nuclear factor in the synovial membrane cells of patients with rheumatoid arthritis^43,44^ and expression of NF-κB p50 and p65 subunits in FLSs under inflammatory stimulus have been reported^45,46^. Our findings that TPCK and SN50, the inhibitors of IK-B phosphorylation and NF-κB translocation and activation, respectively, prevented BaP1-induced expression of COX-2 indicate that activation of NF-κB is a key mechanism by which this metalloprotease triggers COX-2 gene and protein expression and PGE_2_ synthesis in FLSs. These data are supported by the nuclear translocation of NF-κB we observed following stimulation with BaP1, confirming that this metalloprotease can activate NF-κB. In addition, the rapid decrease in non-phosphorylated IκB α levels found in FLSs incubated with BaP1 is in line with the activation of NF-κB by this metalloprotease, demonstrating its stimulatory activity on the signaling pathways that lead to phosphorylation of IκB α and activation of NF-κB. The observation that BaP1 induced translocation of the NF-κB p50 and RelA (p65) subunits into the nucleus gives further support to the hypothesis that this transcription factor is activated by BaP1 and provides a mechanism for this activation. Interestingly, while TPCK treatment reduced mPGES-1 expression, SN50 did not affect BaP1-induced mPGES-1 expression. As SN50 competes with the p50 subunit of NF-κB for the translocation site, it is reasonable to suggest that mPGES-1 expression is not related to the NF-κB p50 subunit and that another subunit may be involved in this effect. To our knowledge, this is the first evidence of activation of NF-κB in FLSs by a metalloprotease and the first evidence of the mechanisms involved in this activation.

A family of EP receptors (EP1- 4) is known to mediate the various biological actions of PGE_2_^47^. In this study, we extended our knowledge of the mechanisms by which BaP1 stimulates FLSs to generate PGE_2_ by focusing on the role of the EP4 prostanoid receptor, which has previously been shown to regulate the actions of PGE_2_ in joint inflammatory conditions^48^ and the increased expression of COX-2 and mPGES-1 in inflammatory cells^4,19^. We found that the EP4 receptor positively regulates BaP1-triggered PGE_2_ release and expression of COX-2 and mPGES-1, which are critically involved in the biosynthesis of PGE_2_ induced by this metalloprotease. These findings are supported by the increased EP4 protein expression following BaP1 stimulation in our experimental model, confirming the ability of this receptor to activate FLSs. Our data agree with previous studies associating increased EP4 expression with inflamed joints in an adjuvant-induced arthritis rat model^49^ as well as in rat and human FLSs stimulated by IL-1β^4,50^. Furthermore, our findings are in line with reports that mammalian MMP-1 and -3 up-regulate EP4 expression in macrophages in culture^19^. To the best of our knowledge, the findings of the present study are the first to show that a metalloprotease can induce expression of this prostanoid receptor in FLSs. It is therefore reasonable to suggest that PGE_2_ engagement of the EP4 receptor triggers a biosynthetic cascade of this mediator, regulating its production through a positive feedback loop. Activation of this loop likely contributes to the increased PGE_2_ levels found throughout the period of stimulation with BaP1 in our study and may explain the absence of a concentration-effect relationship for BaP1-induced PGE_2_ release as the time-course release curve was a plateau. In addition to the EP4 receptor, the EP2 receptor is important in inflammatory processes. Although no information on participation of the EP2 receptor in PGE_2_ release is available in literature, a role of EP2 receptor in the pro inflammatory effect of BaP1 cannot be ruled out.

We have shown a late release of IL-1β in FLSs stimulated by BaP1, a finding consistent with the direct stimulatory activity of BaP1 on FLSs. Moreover, using pharmacological interference we found that IL-1β is involved in the mechanism triggered by BaP1 in FLSs that results in production of PGE_2_. In view of previous reports that IL-1β induces PGE_2_ production via up-regulation of COX-2 and PGESm-1 expression in several cell types, including synovial fibroblasts^51^, it is plausible to suggest that, under the present experimental conditions, IL-1β up-regulates BaP1-stimulated production of PGE_2_ by inducing expression of COX-2 and/or mPGES-1 in FLSs. Taken together, our findings show that the metalloprotease BaP1 directly activates FLSs to produce PGE_2_ by a COX-2-dependent mechanism involving the coupling of the inducible enzymes COX-2 and mPGES-1 and intracellular PLA_2_s. BaP1 also induced release of IL-1β, which up-regulates the production of PGE_2_ at a later stage of the stimulation. Gene and protein expression of both COX-2 and mPGES-1 are induced by BaP1 via activation of distinct subunits of the NF-κB transcription factor. While the NF-κB p50 and p65 subunits are involved in up-regulation of COX-2 expression, only the NF-κB p65 subunit is involved in BaP1-induced mPGES-1 expression. BaP1 also up-regulates EP4 receptor expression. Engagement of this receptor by PGE_2_ triggers a positive feedback loop for production of this mediator by up-regulating key components of the PGE_2_ biosynthetic cascade, such as COX-2, mPGES-1 and the EP4 receptor, thus probably contributing to amplification of the BaP1-induced effect in FLSs. Considered together, these data show that FLSs are an important target for the action of metalloproteases in joint inflammation and provide new insights into the roles of MMPs in inflammatory articular diseases. Additionally, as the BaP1 molecule has only the catalytic domain, our results may give insights into the importance of this domain of MMPs in the inflammatory activities of these enzymes.

## Supplementary information

Supplementary information
