# Supplementary material for: A representative metalloprotease induces PGE2 synthesis in fibroblast-like synoviocytes via the NF-κB/COX-2 pathway with amplification by IL-1β and the EP4 receptor
Source: Sci Rep. 2020 Feb 24;10:3269. doi: 10.1038/s41598-020-59095-z (PMC7039882; doi:10.1038/s41598-020-59095-z)
Supplement: Supplementary file 1 — Supplementary information [file 41598_2020_59095_MOESM1_ESM.pdf]

## Supplementary data

# A representative metalloprotease induces $PGE_2$ synthesis in fibroblast-like synoviocytes via the NF-κB/COX-2 pathway with amplification by IL-1β and the EP4 receptor

Mariana N. Viana[1], Elbio Leiguez Jr.[1], José M. Gutiérrez[2], Alexandra Rucavado[2], Regina P. Markus[3], Marina Marçola[3], Catarina Teixeira[1†], Cristina M. Fernandes[1†*]

[1]Pharmacology Laboratory, Butantan Institute, São Paulo, SP, Brazil; [2]Clodomiro Picado Institute, School of Microbiology, University of Costa Rica, San José, Costa Rica; [3]Department of Physiology, Institute of Biosciences, São Paulo University, São Paulo, SP, Brazil

[†] These authors contributed equally to this work

***Corresponding author:** Cristina Maria Fernandes, Pharmacology Laboratory, Butantan Institute, Avenida Vital Brasil 1500, 05503-900, São Paulo, SP, Brazil. Tel: +55 11 37232150, Fax: +55 11 26279752. E-mail: cristina.fernandes@butantan.gov.br

**Supplementary Figure – 1 Fernandes**

Cytotoxicity to FLSs of BaP1

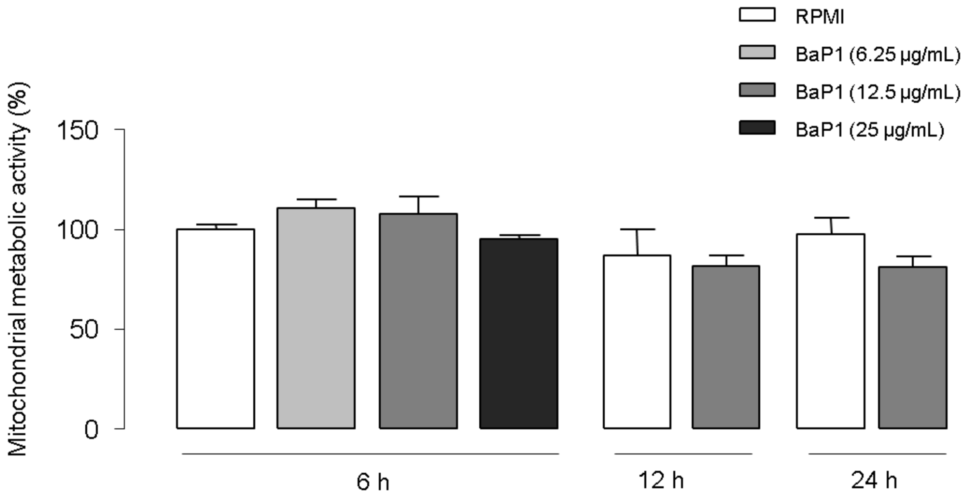

**Supplementary Figure – 2 Fernandes**

Immunostaining of CD90 was performed to detect FLSs.

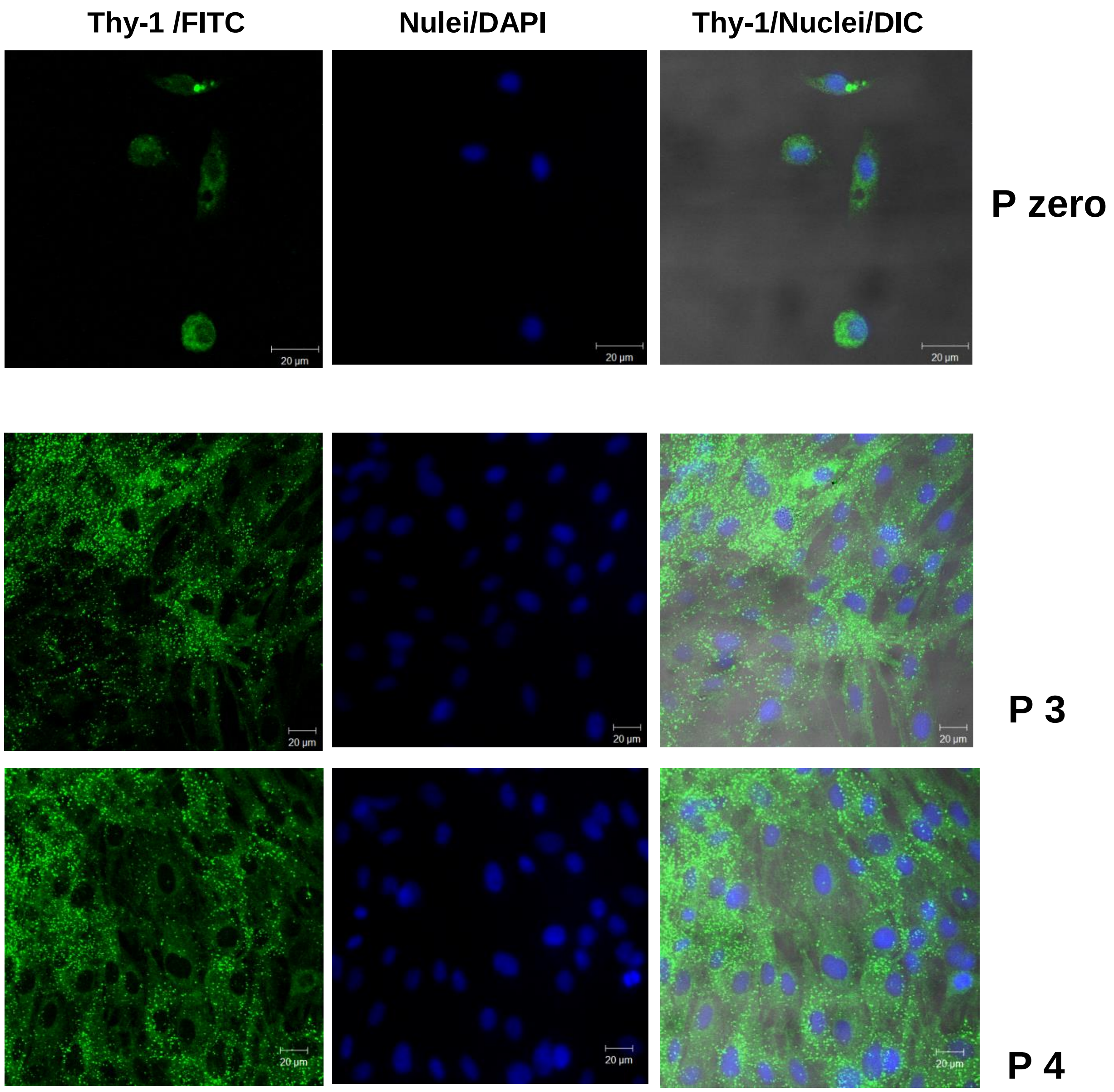

## Supplementary Figure – 3 Fernandes

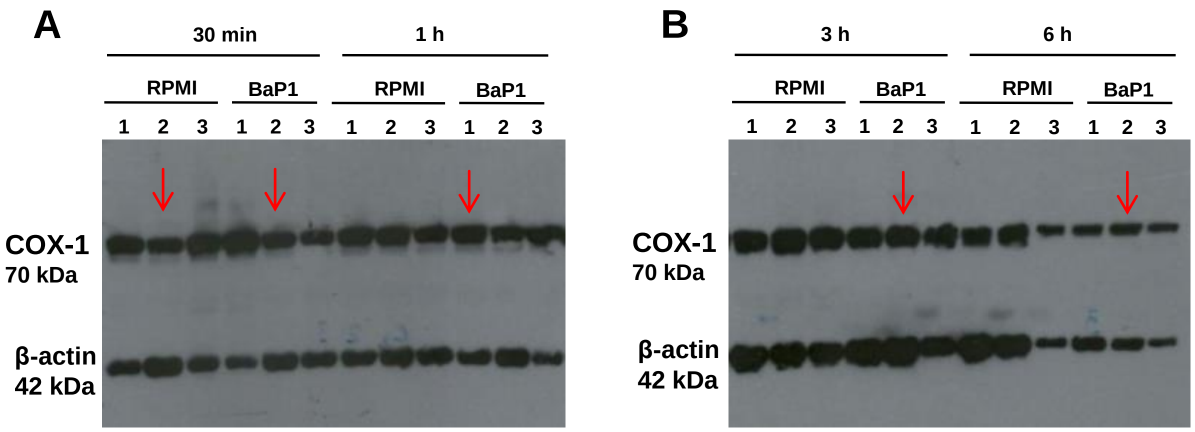


## Supplementary Figure – 4 Fernandes

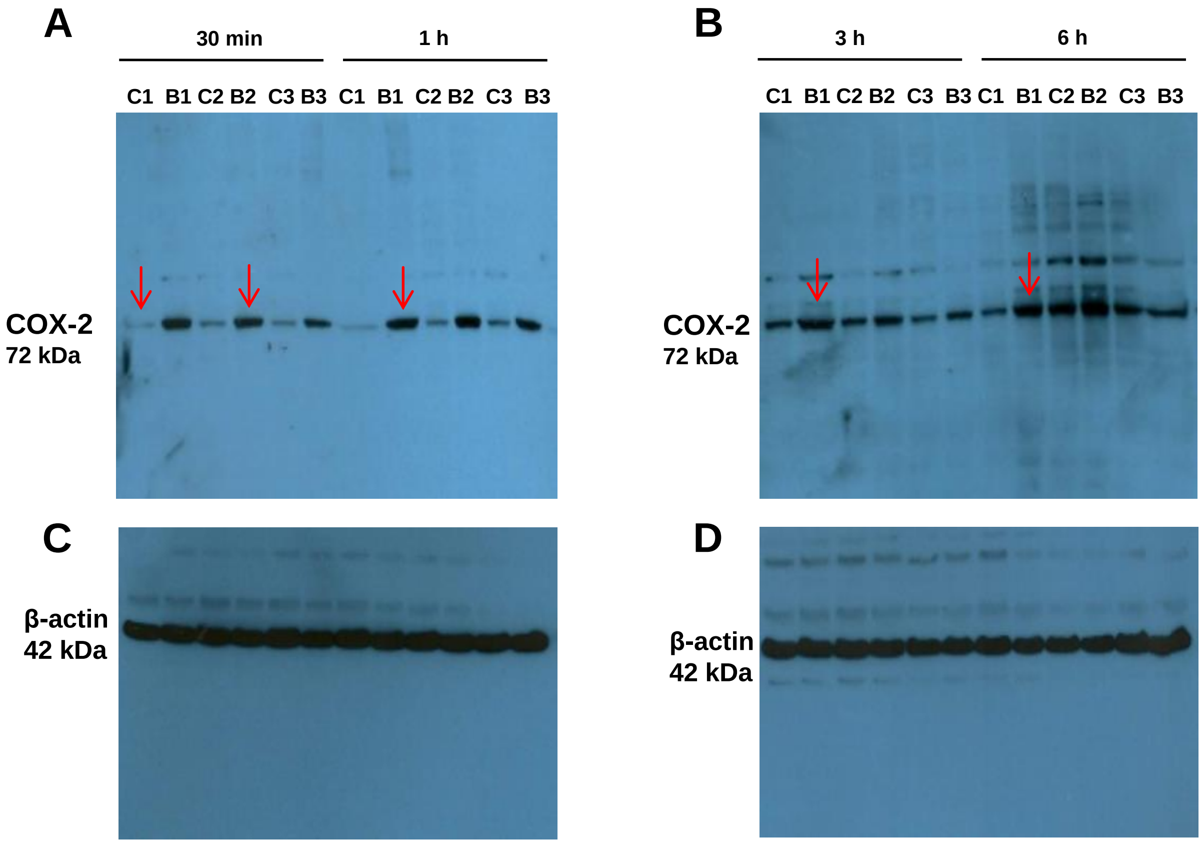

**Supplementary Figure – 5 Fernandes**

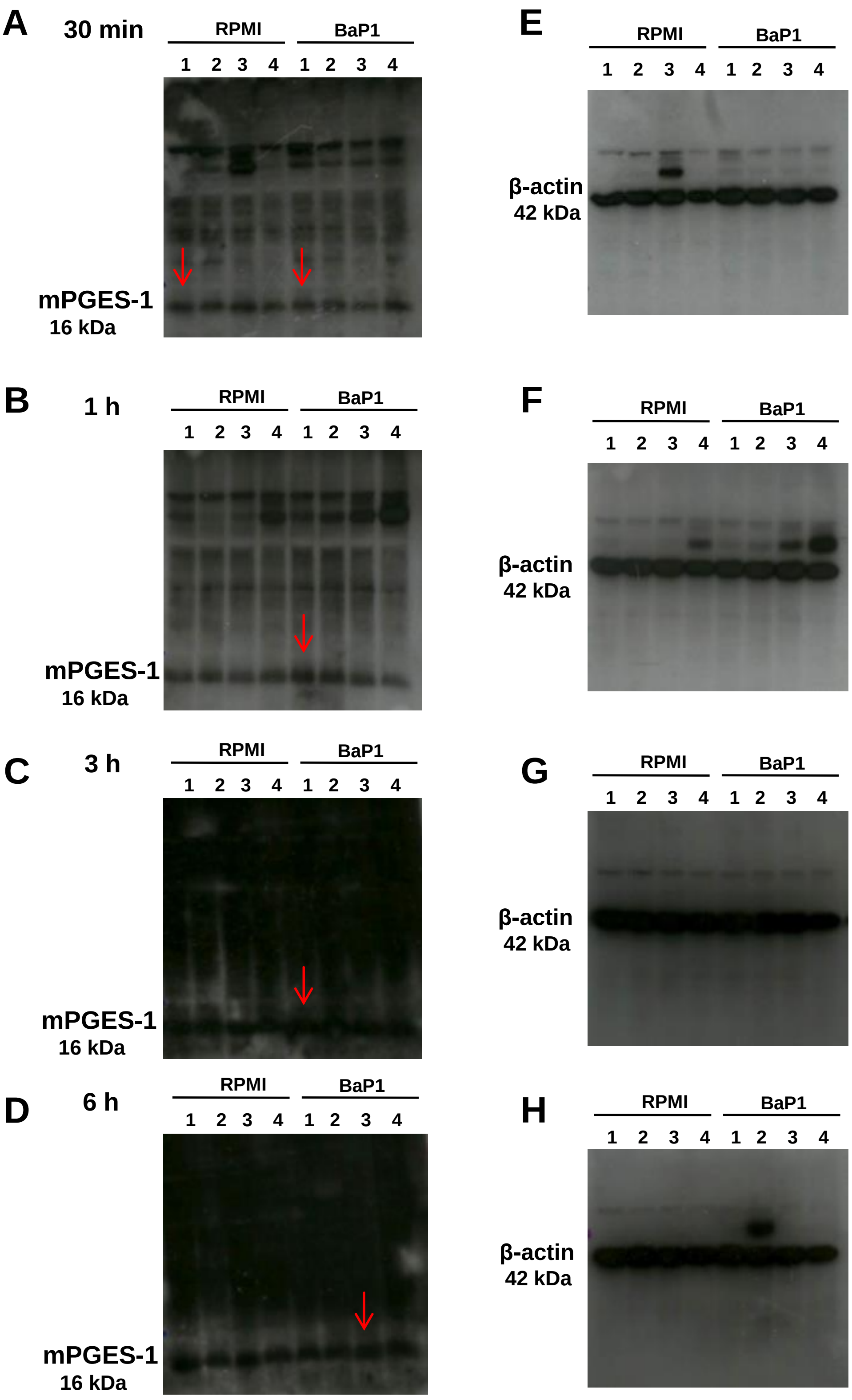

**Supplementary Figure – 6 Fernandes**

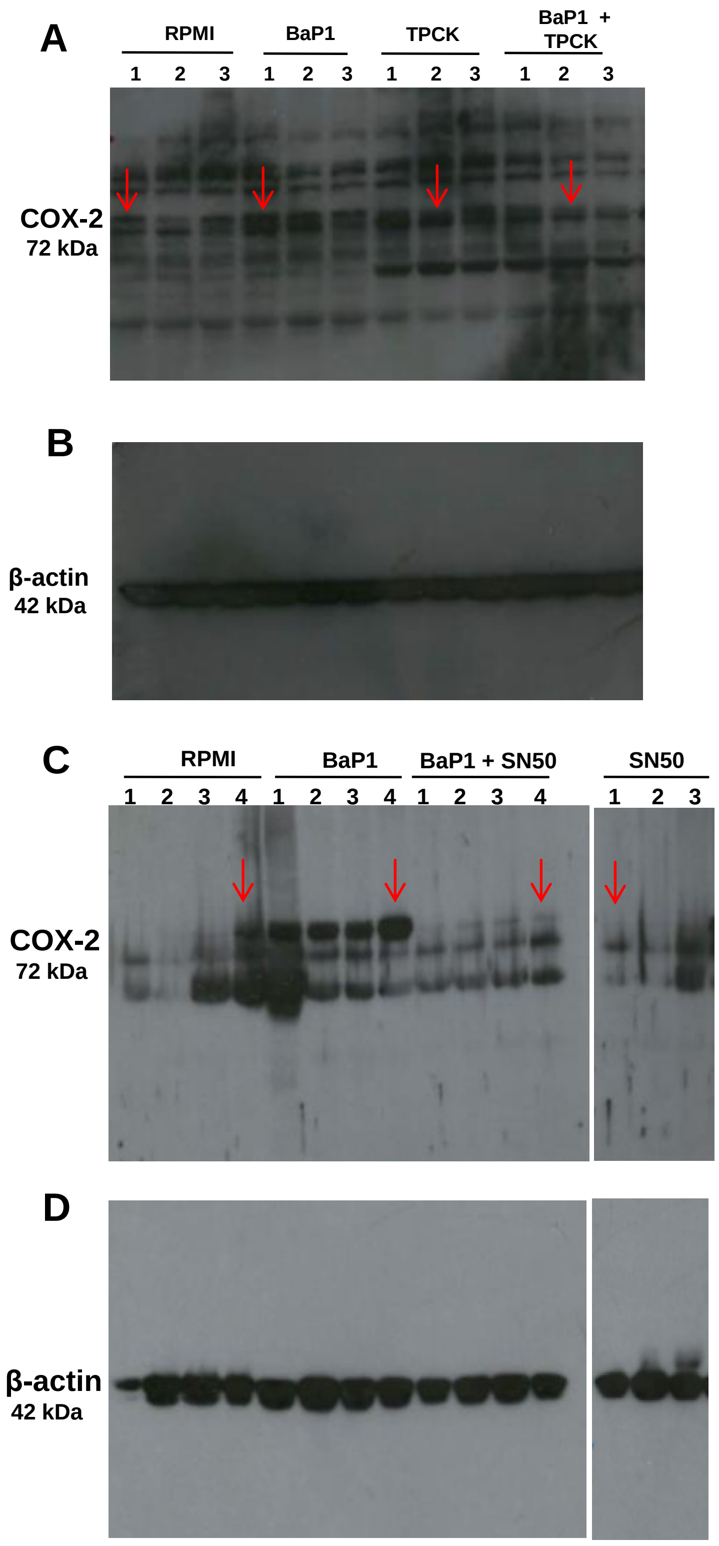

## Supplementary Figure – 7 Fernandes

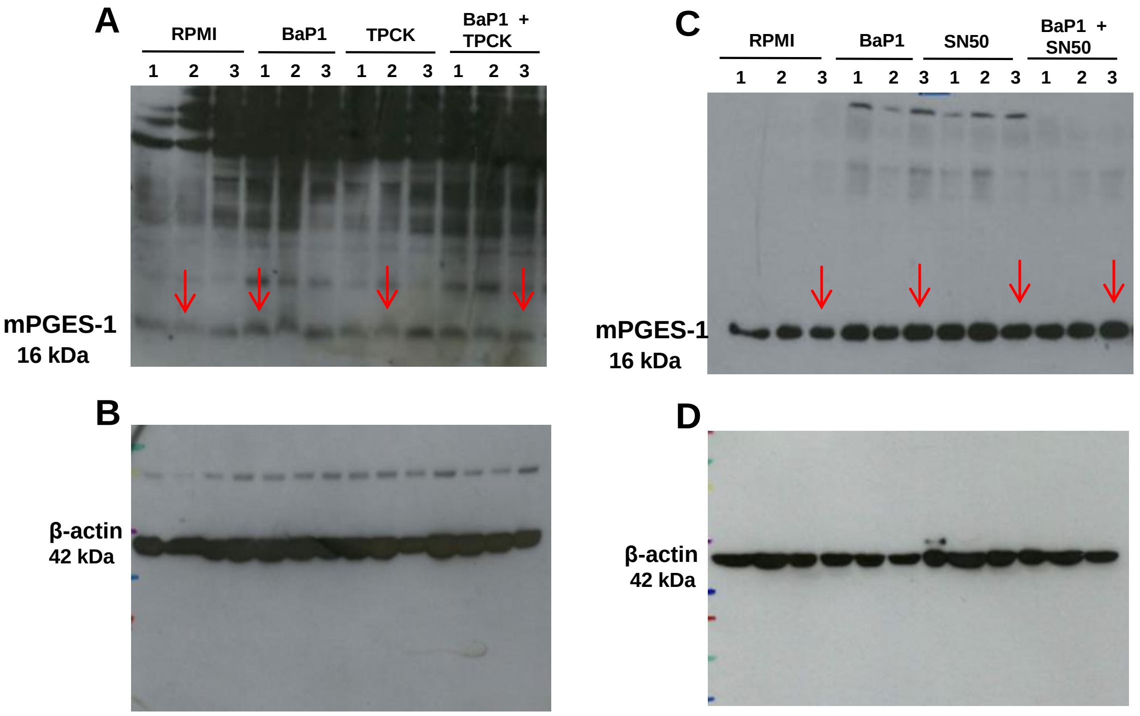


## Supplementary Figure – 8 Fernandes

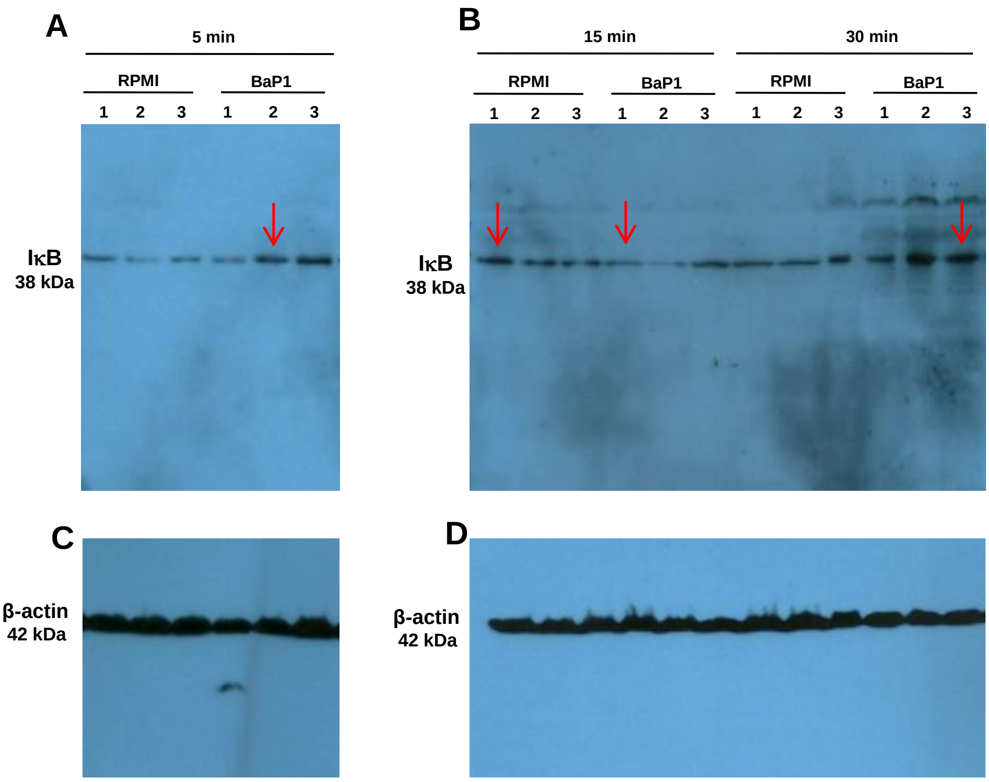

Supplementary Figure – 9 Fernandes

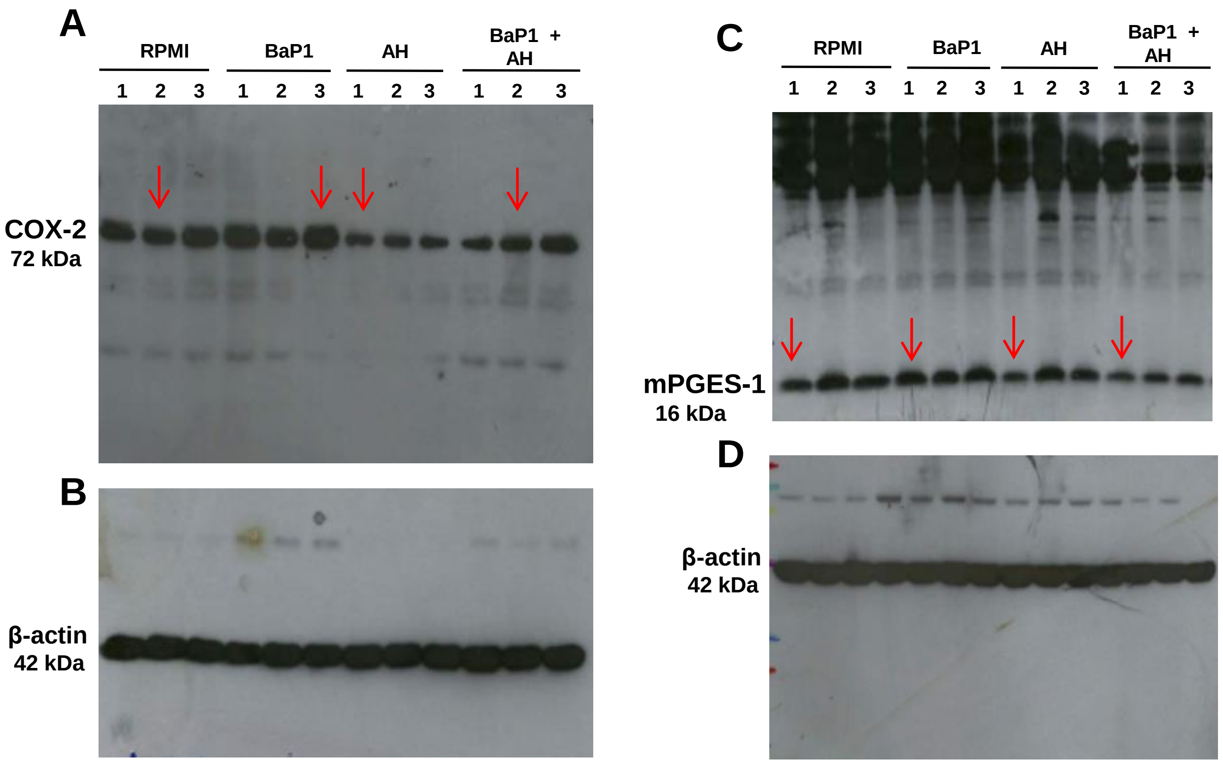


Supplementary Figure – 10 Fernandes

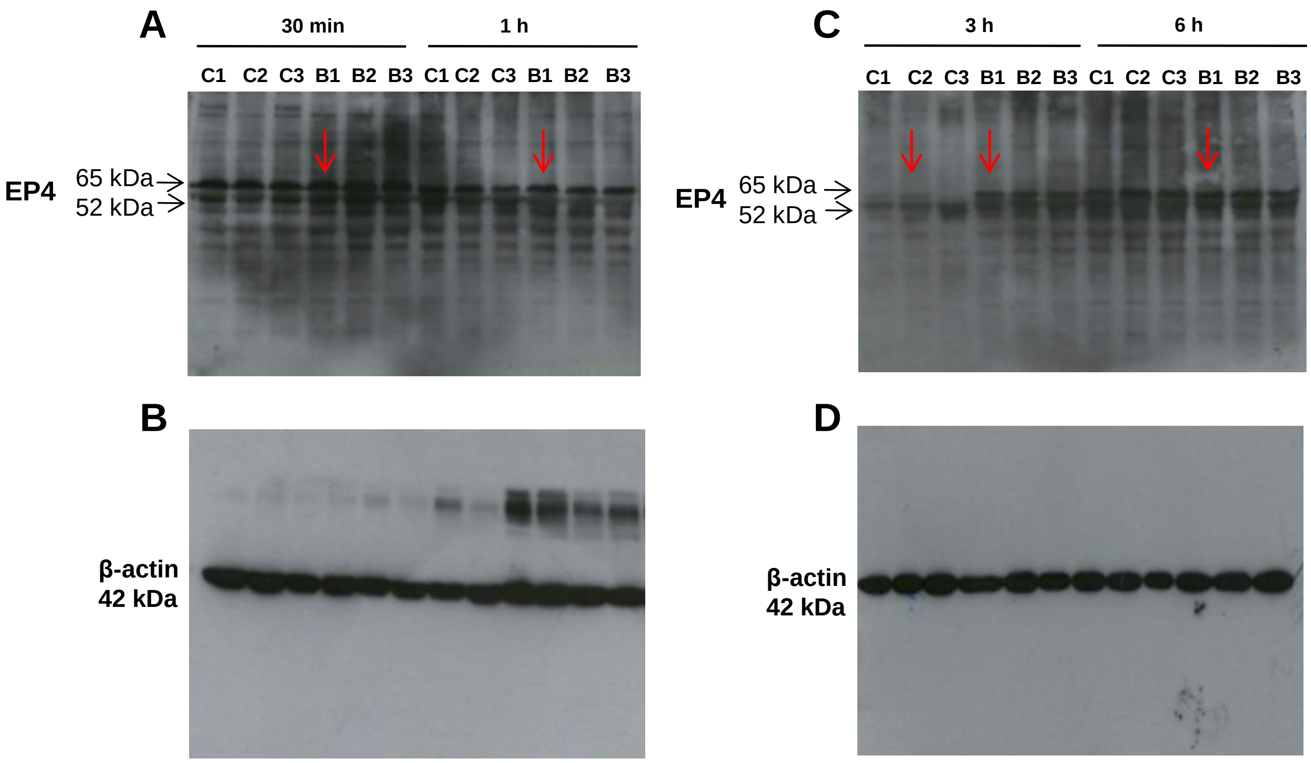

**Supplementary Figure 11 – Fernandes**

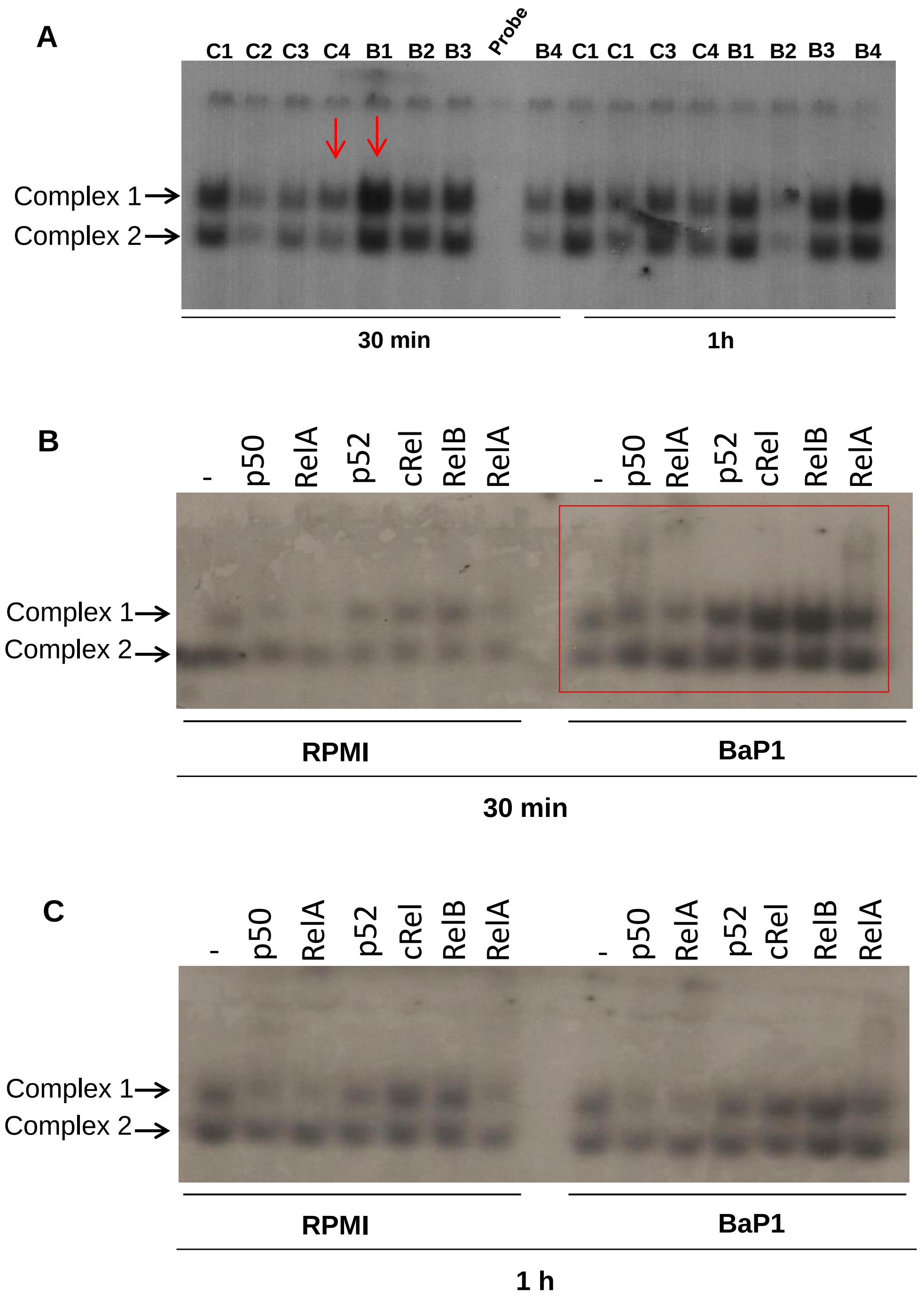

**Supplementary Figure 1. Effect of different concentrations of BaP1 on cell viability.** FLSs were incubated with BaP1 (6.25, 12.5 and 25 μg/mL) or RPMI (control) for 6h, 12 h and 24h, and cytotoxicity was assessed by the tetrazolium-based (MTT) colorimetric assay. Values represent the mean ± SEM of five samples. *p<0.05 relative to control (RPMI). # p<0.05 relative to 3.125 μg/mL.

**Supplementary Figure 2. Immunocytochemistry to detect Thy-1 using fluorescein isothiocyanate [FITC] in fibroblast-like synoviocytes isolated from rat joints**. Cells from rat synovial membrane were adhered to glass slides and incubated in RPMI for 48 h. Nuclei were stained with DAPI. P zero = non-passaged cells; P3 = third passage; P4 = fourth passage.

**Supplementary Figure 3. BaP1 do not induces COX-1 protein expression in FLSs.** Isolated FLSs were incubated with BaP1 (12.5 μg/mL) or RPMI (control) for 30 min, 1, 3 and 6 h. (A) Immunoreactive bands of COX-1 and β-actin (loading control), 30 min and 1 h of incubation with BaP1 and (B) Immunoreactive bands of COX-1 and β-actin (loading control), 3 and 6 h of incubation with BaP1.

**Supplementary Figure 4. BaP1 up-regulates COX-2 protein expression in FLSs.** Isolated FLSs were incubated with BaP1 (B) (12.5 μg/mL) or RPMI (C) (control) for 30 min, 1, 3 and 6 h. (A) Immunoreactive bands of COX-2, 30 min and 1 h after incubation of FLSs with BaP1 and (B) Immunoreactive bands of COX-2 after 3 and 6 h of incubation. (C) Immunoreactive bands of β-actin (loading control), 30 min and 1 h after incubation of FLSs with BaP1 and (D) Immunoreactive bands of β-actin (loading control), 3 and 6 h after incubation.

**Supplementary Figure 5. BaP1 up-regulates mPGES-1 protein expression in FLSs.** Isolated FLSs were incubated with BaP1 (12.5 μg/mL) or RPMI (control) for 30 min, 1, 3 and 6 h. Western blotting of mPGES-1 and β-actin (loading control) in FLSs extracts. (A-D) Full length blots of mPGES-1 and (F-H) Full length blots of β-actin.

**Supplementary Figure 6. NF-kB transcription factor is relevant to BaP1-induced COX-2 protein expression by FLSs.** Isolated FLSs were pretreated with TPCK (25 μM) or SN50 (50 μg/mL) for 1 hour and stimulated by BaP1 (12.5 mg/mL) or RPMI (control) for 3 h. (A) COX-2 immunoreactive bands of cells pretreated with TPCK and (C) COX-2 immunoreactive bands of cells pretreated with SN50. (B and D) Immunoreactive bands of β-actin (loading control).

**Supplementary Figure 7. NF-kB transcription factor is relevant to BaP1-induced mPGES-1 protein expression by FLSs.** Isolated FLSs were pretreated with TPCK (25 μM) or SN50 (50 μg/mL) for 1 hour

and stimulated by BaP1 (12.5 mg/mL) or RPMI (control) for 3 h. (A) mPGES-1 immunoreactive bands of cells pretreated with TPCK and (C) mPGES-1 immunoreactive bands of cells pretreated with SN50. (B and D) Immunoreactive bands of β-actin (loading control).

**Supplementary Figure 8. BaP1 down-regulates IkB protein expression in FLSs.** Isolated FLSs were incubated with BaP1 (12.5 µg/mL) or RPMI (control) for 5, 15, and 30 min. Western blotting of IkB and β-actin (loading control) in FLSs extracts. (A and B) Full length blots of IkB and (C and D) Full length blots of β-actin.

**Supplementary Figure 9. EP4 receptor antagonist blocks BaP1-induced COX-2 and mPGES-1 expression in FLSs.** Isolated FLSs were pretreated with AH23848 (30µM) and stimulated by BaP1 (12.5 mg/mL) or RPMI (control) for 3 hours. (A) COX-2 immunoreactive bands of cells pretreated with AH23848 and (C) mPGES-1 immunoreactive bands of cells pretreated with AH23848. (B and D) Immunoreactive bands of β-actin (loading control).

**Supplementary Figure 10. BaP1 up-regulates EP4 receptor protein expression in FLSs.** Isolated FLSs were incubated with BaP1 (B) (12.5 µg/mL) or RPMI (C) (control) for 30 min, 1, 3 and 6 h. Western blotting of EP4 receptor and β-actin (loading control) in FLSs extracts. (A and C) Full length blots of EP4 receptor and (B and D) Full length blots of β-actin.

**Supplementary Figure 11**. **Effect of BaP1 on NF-kB activity in FLSs.** Cultured FLS were exposed to BaP1 (B) (12.5 µg/mL) or RPMI (C) (Control) for 30 and 60 min. Nuclear extracts were then prepared and analyzed by electrophoretic mobility shift assay (EMSA). (A) Immunoreactive bands of NF-kB (complex 1 and 2). (B) Immunodetection of NF-kB subunits -p50, RelB, p52, cRel and RelA- after 30 min of incubation with BaP1 and (C) Immunodetection of NF-kB subunits -p50, RelB, p52, cRel and RelA- after 1 h of incubation with BaP1 (supershift assay).
